# Percolation Effects in Mixed Matrix Membranes with Embedded Carbon Nanotubes

**DOI:** 10.3390/membranes12111100

**Published:** 2022-11-04

**Authors:** Yury Eremin, Alexey Grekhov, Anton Belogorlov

**Affiliations:** 1 Molecular Physics Department, National Research Nuclear University Moscow Engineering Physics Institute, Kashirskoe Highway 31, 119991 Moscow, Russia; 2 A.V. Topchiev Institute of Petrochemical Synthesis, Russian Academy of Sciences, Leninsky Prospekt, 29, 119991 Moscow, Russia; 3 Research Institute for Graphite-Based Structural Materials “NIIgrafit” (JSC “NIIgrafit”), 111524 Moscow, Russia

**Keywords:** CNT, percolation, mixed matrix membrane

## Abstract

Polymeric membranes with embedded nanoparticles, e.g., nanotubes, show a significant increase in permeability of the target component while maintaining selectivity. However, the question of the reasons for this behavior of the composite membrane has not been unequivocally answered to date. In the present work, based on experimental data on the permeability of polymer membranes based on Poly(vinyl trimethylsilane) (PVTMS) with embedded CNTs, an approach to explain the abnormal behavior of such composite membranes is proposed. The presented model considered the mass transfer of gases and liquids through polymeric membranes with embedded CNTs as a parallel transport of gases through the polymeric matrix and a “percolation” cluster—bound regions around the embedded CNTs. The proposed algorithm for modeling parameters of a percolation cluster of embedded tubular particles takes into account an agglomeration and makes it possible to describe the threshold increase and subsequent decrease permeability with increasing concentration of embedded particles. The numerical simulation of such structures showed: an increase in the particle length leads to a decrease in the percolation concentration in a matrix of finite size, the power of the percolation cluster decreases significantly, but the combination of these effects leads to a decrease in the influence of the introduced particles on the properties of the matrix in the vicinity of the percolation threshold; an increase in the concentration of embedded particles leads to an increase in the probability of the formation of agglomerates and the characteristic size of the elements that make up the percolation cluster, the influence of individual particles decreases and the characteristics of the percolation transition determine the ratio of the sizes of agglomerates and matrix; and an increase in the lateral linear dimensions of the matrix leads to a nonlinear decrease in the proportion of the matrix, which is affected by the introduced particles, and the transport characteristics of such MMMs deteriorate.

## 1. Introduction

The addition of nanoparticles into polymers is one of the methods for obtaining nanocomposite materials with new functional properties, which are determined both by the properties of the particles and the polymer and by the structural characteristics of clusters of embedded particles [1,2]. Carbon nanotubes (CNTs) are one of the most promising types of nanoparticles, which, due to the unique variety of geometric, structural, and physical characteristics, make it possible to obtain composite materials with a wide range of changes in properties from structural, strength, and electromagnetic, to optical. Of particular interest is the experimentally-confirmed significant change in the transport and selective properties of liquids and gases in polymeric membrane materials with CNTs. The properties of such mixed matrix membrane (MMM) materials allow the solving of the unique problems of separation and purification of gases and liquids in petrochemistry, medicine, food production, processing of household, and industrial waste, etc.

Changes in the transport properties of polymers with increasing CNTs concentration in the simplest case are proportional to the CNTs concentration. In this case, both an improvement in properties and a decrease in the permeability of materials are possible, which is associated with the influence of polymer regions modified due to interaction with CNTs. For example, when CNTs with concentrations of 0, 1, 2.5, 5, 10, and 15 wt.% are introduced into poly(bisphenol A-co-4-nitrophthalic anhydride-co-1,3-phenylene diamine) (PBNPI), the permeability of various gases (H_2_, CO_2_, and CH_4_) increases without compromising selectivity [3]. The introduction of CNTs with concentrations of 0.5 and 2% leads to an increase in the permeability of water through poly(vinyl alcohol) (PVA); the authors attribute this effect to an increase in the hydrophilicity of the polymer regions near the CNTs [4]. The mass transfer of a water/ethanol mixture in the mode of pervaporation through PVA increases with an increase in the concentration of CNTs [5]. As the authors explain, a strong interaction between the polymer and CNTs leads to a decrease in the mobility of polymer chains and the degree of swelling. Also, the observed monotonic increase in the permeability coefficient is explained by the possible transport of molecules along the internal channel of the CNT. A deterioration of permeability was found for polydimethylsiloxane (PDMS) samples with CNTs concentrations from 0 to 10% (wt.) during the transport of air mixtures with different contents of H_2_ and CH_4_. The authors attribute this increase in the diffusion path to an increase in the energy of interaction between CH_4_ molecules and a polymer with embedded CNTs [6]. The water flow in polyethylene glycol (PEG) with a molecular weight of 10,000 (PEG10000) decreased with increasing CNTs concentration from 0 to 7 wt.%, which the authors attributed to a decrease in the polymer pore size in the vicinity of the CNTs surface (pore blocking effect) [7].

However, in a significant number of experiments, a nonlinear change in the transport properties of MMMs is observed, and the permeability coefficient increases at low CNTs concentrations and stops changing or decreases when a certain critical concentration is reached [8,9,10,11,12]. For example, in the polymers of intrinsic microporosity (PIM) PIM-1 polymer, the permeability of O_2_, N_2_, and CH_4_ at a CNTs concentration from 0 to 2% increased by 80%, 29%, and 193%, respectively, but at a concentration of 3% it decreased for these gases. At the same time, for CO_2_, the permeability at a CNTs concentration from 0 to 2% increased by 54%, while at a concentration of 3% it remained unchanged [11]. The authors explained this result by a decrease in the permeability coefficient associated with CNTs agglomerations. In PEG6000/CNTs with concentrations from 1 to 13% (wt.), the water flow increased up to a concentration of 10% and then decreased. The authors explain this effect by the formation of a CNTs network upon reaching a certain concentration, which connects the polymer pores, and a further decrease occurs during CNTs agglomeration [7]. When CNTs were introduced into PVA at concentrations of 0.5 to 2.5% (wt.), the increase in the permeability of the benzene/cyclohexane mixture increased at concentrations up to 2%, leading to a threefold increase in permeability, and a further increase in CNTs concentration leads to a decrease in permeability. The authors attribute this effect to a change in the free volume through which mass transfer occurs [13]. Similar effects were observed in experiments with other polymers [14,15,16,17]. Recent studies of the CNTs introduction into the polymers report the overcoming of the Robeson upper bound [18,19,20,21]. For example, the addition of 0.5% surface-engineered multi-walled carbon nanotubes into the PVA/PEG nano-composite membranes shows maximum CO_2_ permeability and CO_2_/N_2_ and CO_2_/CH_4_ selectivity [18]. In another work [19], CO_2_ permeability was increased up to 369.1 barrer with CO_2_/N_2_ selectivity of 110.8 for a hybrid CNTs-PEG membrane containing 3 wt.% of CNTs. The different additives, functional groups (–COOH, –NCO, and –NH_2_) on the surface of multi-walled carbon nanotubes (MWCNTs), which were then incorporated as fillers in the poly(ether-block-amide) (PEBA) polymeric matrix, also improved gas selectivity and permeability [20]. A similar result was observed for the introduction of non-covalently-functionalized MWCNTs by poly(styrenesulfonate) (PSSA) and poly(vinylpyrrolidone) (PVP) into the poly(vinyl alcohol) matrix [21].

There is no single approach to explaining such nonlinear changes in MMM properties. There are two common approaches. In self-consistent models (for example, Maxwell, Cahn-Jones-Nair et al. [22]), the influence of individual particles on a material is considered without taking into account cooperative effects in the interaction between nanoparticles. The second approach is based on the ideas of percolation theory (for example, the models of Shen, Kripatrik, et al. [23], which take into account the interaction of nanoparticles and consider the cooperative effect on the properties of the material of bound clusters of nanoparticles. However, neither of these approaches explains the nonlinear changes in the transport properties of MMMs and generally do not take into account the size effects of embedded structures in such materials. However, most MMMs with CNTs, in which the characteristic size of the selective layer is comparable to the size of CNTs, belong to such systems, and the influence of size and surface effects on the bulk properties of the material becomes significant.

In our work, we propose a model for describing the transport properties of MMMs that takes into account the dimensional characteristics of the percolation transition, taking into account the interfacial interaction, the geometric characteristics of CNTs, and the parameters of the polymer matrix. For the ideal case of individual particles in a matrix, we have shown that the ratio of membrane and particle sizes determines the concentration of percolation cluster formation and its parameters. The obtained results of direct numerical simulation of the parameters of a percolation cluster make it possible to describe the entire set of known changes in transport properties and to estimate the parameters of CNTs in a polymer. The proposed approach makes it possible to describe the nonlinear change in the permeability of polymers when CNTs are introduced into them.

## 2. Calculation

An experimental study of the influence of the parameters of embedded nanoparticles on the transport characteristics of polymers was carried out for a group of gases for MMMs based on Poly(vinyl trimethylsilane) (PVTMS) with embedded CNTs [24]. Similar results were obtained for MMMs based on Poly(methyl methacrylate) (PMMA) with embedded CNTs [25].

Since CNTs with a large aspect number were used in these experiments, to describe the observed effects, models were used in which the particle form factor is taken into account when calculating the permeability: Maxwell−Wagner−Sillar [26], Petropoulous and Toy [27], Kang−Jones−Nair [22]. In the Maxwell−Wagner−Sillar model, the concentration of ellipsoids (1) is calculated, which at *n* = 1 is converted into a classical model of a series connection of layers, at *n* = 0 into a classical model of a parallel connection of layers, *n* = 1/3 into a Maxwell model.

(1) $$P_{eff}=P_{c}\frac{nP_{d}+\left(1-n\right)P_{c}-\left(1-n\right)\varphi_{d}\left(P_{c}-P_{d}\right)}{nP_{d}+\left(1-n\right)P_{c}+n\varphi_{d}\left(P_{c}-P_{d}\right)}$$

where *P_eff_* is the effective permeability coefficient of the MMM, *P_c_* is the permeability coefficient of the continuous (polymer) phase, *P_d_* is the permeability coefficient of the dispersion (nanoparticle) phase, *φ_d_* is the volume fraction of the dispersion phase, and *n* is a particle shape factor, which depends on the long-to-short-axis-length ratio. For oblate ellipsoids (the longest axis is aligned with the permeation direction), 0 < *n* < 1/3; for oblate ellipsoids (the shortest axis is aligned with the permeation direction) 1/3 < *n* < 1 [26].

In the Petropoulous and Toy model, systems with particles of various shapes are considered.

(2) $$P_{eff}=P_{c}\left[1+\frac{\left(1+G\right)\varphi_{d}}{\left(\frac{P_{d}/P_{c}+G}{P_{d}/P_{c}-1}\right)-\varphi_{d}}\right]$$

where *G* is a geometric factor accounting for the effect of dispersion shape. *G* equals 1 for long and cylindrical (elongated) particles dispersed transverse to the gas flow direction. *G* is 2 for spherical particles or isometric aggregates. In the case of planar (laminate) particles, *G* tends to infinity if the dispersed particles are oriented in lamellae parallel to the gas flow direction, minimizing resistance to flow. Conversely, *G* tends to zero if the dispersed particles are oriented in lamellae perpendicular to the gas flow direction, maximizing the impedance of flow [27].

The Kang-Jones-Nair model describes tubular particles and the equation for concentration (3) takes into account not only the permeability of the dispersed phase but also the orientation of tubular particles

(3) $$\frac{P_{eff}}{P_{c}}={\left[\left(1-\frac{cos\theta }{cos\theta +\frac{1}{\alpha }sin\theta }\varphi_{f}\right)+\frac{P_{c}}{P_{d}}\left(\frac{1}{cos\theta +\frac{1}{\alpha }sin\theta }\right)\varphi_{d}\right]}^{-1}$$

where α = l/*d* is the aspect ratio of tubular fillers and *θ* is the filler orientation angle with respect to the membrane transport direction, varying from 0 to π/2 radians.

**Figure 1:**
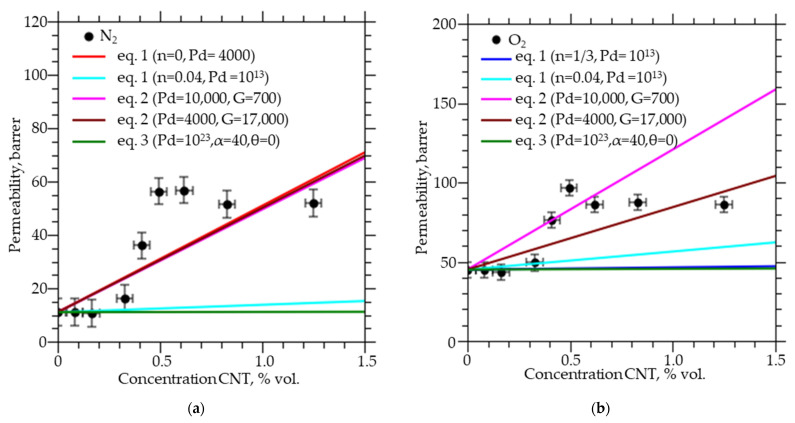

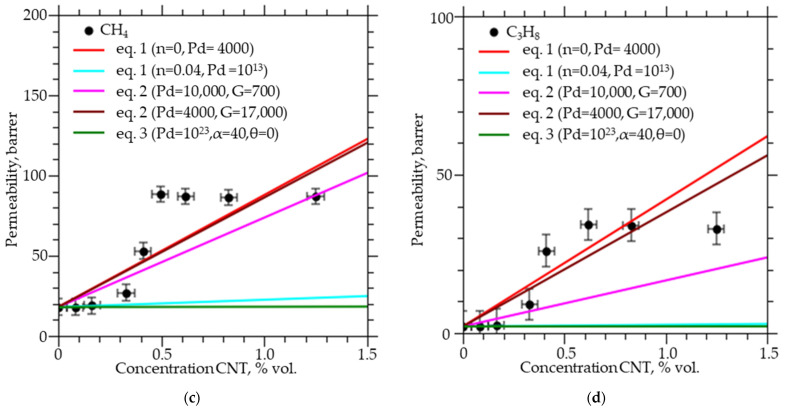
Permeability coefficients of (**a**) nitrogen, (**b**) oxygen, (**c**) methane, and (**d**) propane through PVTMS membranes with different CNTs concentrations and calculation by models.

Figure 1 shows the experimental data and calculation by classical models on the permeability of gases (N_2_, O_2_, CH_4_, and C_3_H_8_) through PVTMS with a CNTs concentration of up to 1.5% (mass). It can be seen that for all gases there is a sharp increase in permeability at CNTs concentrations from 0.3 to 0.5% and a slight change in permeability with a further increase in concentration. Similar results were obtained in a number of works [8,9,10,11,12,14,15,16,17,18,19,20,21].

By the Maxwell−Wagner−Sillar model with a dimension factor of *n* = 0 (parallel connection model) and a CNTs permeability coefficient of 4000 barrer for nitrogen and oxygen and 7000 barrer for methane and propane, it is possible to describe for all gases an increase in the permeability coefficient at a volume concentration of CNTs from 0 to 1.5% by several times, but the gas permeability coefficient linearly depends on the concentration of CNTs, and this does not allow us to describe the absence of the effect of the introduction of CNTs at CNTs concentrations from 0 to 0.3%, and the absence of an increase in the permeability coefficients with increasing concentration from 0.6 to 1.3%. At *n* = 1 (serial connection model), 1/3 (Maxwell model), 0.04 (prolonged ellipsoids) and permeability coefficient for all gases from 1 to 1013 barrer, the change in gas permeability coefficients observed in the experiment could not be described. In calculations according to the Petropoulous and Toy model, the geometry factor varied from 1 to 17,000, and the permeability coefficient varied from 1 to 100,000 barrer, this model allows describing the increase in the gas permeability coefficient at CNTs concentrations from 0 to 1.5%, at *G* = 17,000 and *P_d_* = 4000 barrer for nitrogen, oxygen, and propane and *P_d_* = 7000 barrer for methane. The model of Petropoulous and Toy does not allow describing the absence of the effect from the introduction of CNTs at CNTs concentrations from 0 to 0.3%, and the absence of an increase in permeability coefficients with an increase in concentration from 0.6 to 1.3%, since at CNTs concentrations from 0 to 1.5% The gas permeability coefficients calculated using this model depend linearly on the CNTs concentration. The Kang-Jones-Nair model with permeability coefficients of a dispersed medium, in our case, CNTs, from 1 to 1023 and α from 1 to 40, describes the changes in gas coefficients observed in the experiment only at CNTs concentrations from 0 to 0.3%.

All the models used, within the limits of experimental error, can describe the linear areas of permeability change, but at the same time, the values of the permeability of the filler lose their physical meaning, because they take too large values. However, the threshold change upon reaching the critical concentration cannot be explained within these models (Figure 1). In recent works [28,29,30,31,32,33] two types of description have been proposed. One [28,29,30,31] is the numerical solution of the three-dimensional Fick’s diffusion equation using the finite differences method or the resistance-based models under the assumption of an ideal morphology. The second [32,33] is an assumption of non-ideal morphology and the influence of the interfacial layer. However, the authors do not consider the formation of bound regions, so their models do not describe a dataset where the permeability of polymers from CNTs concentration varies non-linearly and the effect is observed in a narrow range of concentrations.

To describe the observed threshold change, it is necessary to take into account the parameters of interconnected structures of individual particles, which, when a certain concentration is reached, form a percolation cluster. In such systems, mass transfer through regions of three types is possible: the initial polymer; modified regions of the polymer at the interface with CNTs; and transport through internal cavities in CNTs. In the simplest case, one can consider independent transport through the regions of the unmodified polymer and transport through the percolation cluster from the regions of modified CNTs, the fraction of which in the membrane volume is $C_{x}$:

(4) $$K_{MMM}=K_{x}\cdot C_{x}\left(L,R,dR\right)+K_{p}\cdot \left(1-C_{x}\left(L,R,dR\right)\right)$$

where $K_{x}\;$ is the permeability coefficient of the percolation cluster, $K_{p}$ is the permeability coefficient of the polymer, and *dR* is the thickness of the interfacial layer. The $C_{x}$ value changes non-linearly when the concentration of nanotubes exceeds the percolation “threshold”, the value of which is determined both by the geometrical parameters of the membrane and particles and by the interaction of the polymer with the CNT surface. At concentrations below the “threshold”, the percolation cluster is not formed, and transport over the membrane regions containing CNTs can be neglected.

**Figure 2:**
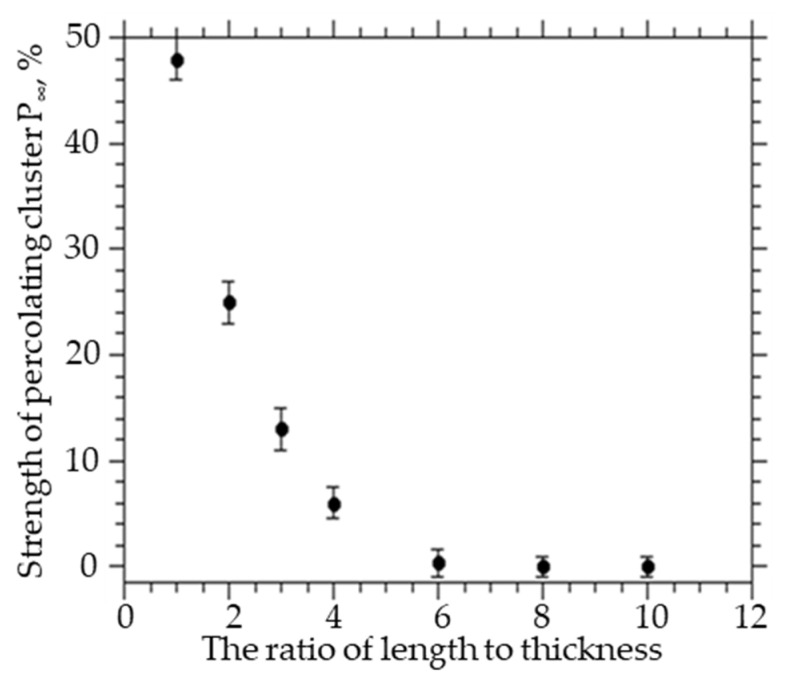
The strength of the percolation cluster at different ratios of lateral dimensions to the thickness of the matrix.

For most of the considered MMMs, the CNTs size is comparable to the thickness of the selective layer or membrane, and it is necessary to take into account the dispersion of the CNTs threshold concentration in systems of finite size. To determine the parameters of a percolation cluster of CNTs in systems of finite size, a software package was developed and the parameters of the percolation transition were modeled in a wide range of membrane and CNTs sizes and particles with different size and shape distributions: sphere, sphere with an impermeable core, spherocylinder, and spherocylinder with an impermeable core, etc. Let us consider the algorithm for calculating the parameters of a percolation cluster using the example of spherocylinders with impermeable cores (Figure 2). Each spherocylinder is randomly placed in space (using the pseudo-random number generator “Mersenne vortex” [34]) and surrounded by a layer (shell) of a modified polymer, thickness *dR* = *R* − *R*_0_. When the shells intersect, a channel (cluster) is formed, which, when the opposite faces of the matrix are connected, forms a percolation cluster. For each concentration of CNTs, 1000 iterations were carried out. By varying the geometrical parameters of the matrix, particles, shell, and the number of particles, it is possible to determine the parameters of emerging clusters and percolation conditions.

For various parameters of particles and matrix, the volume concentration of particles was determined:

(5) $$C_{x}=\left(\frac{4\cdot \pi }{3}\cdot R_{0}^{3}+\pi \cdot R_{0}^{2}\cdot L\right)\cdot N/V$$

where *R*_0_ is the radius of the impermeable core, *L* is the length of the impermeable core, *N* is the number of capsules, and *V* is the volume of the matrix. The percolation cluster power is:

(6) P∞=V∞V

where $V_{\infty }$ is the volume fraction of particles in the percolation cluster and *V* is the volume fraction of all added spherocylinders. The average value of the volume fraction occupied by high-permeability regions of the polymer is:

(7) $$C_{x}=\left(\frac{4\cdot \pi }{3}\cdot (R^{3}-R_{0}^{3})+\pi \cdot (R^{2}-R_{0}^{2})\cdot L\right)\cdot \frac{N_{X}}{V}$$

where $N_{X}\;$ is the number of capsules in the percolation cluster. To verify the algorithm and program, the percolation threshold was calculated for various geometric objects as presented in Table 1 (the data have been rounded to 3 decimal places).

## 3. Result and Discussion

To study the influence of the geometric dimensions of CNTs (length) on the parameters of the percolation cluster, calculations were carried out for spherocylinders with an inner radius *R*_0_ = 0.025 and an outer radius *R* = 0.125 in a cubic system of dimensions 25 × 25 × 25, their volume concentration varied from 0.1 to 0.5% (the number of spherocylinders from 2000 to 50,000), the length varied from 2 to 5. An increase in the CNTs length by 2.5 times leads to an inversely proportional decrease in the CNTs concentration by a factor of 2 (from 0.4 to 0.2%), at which a percolation cluster is formed with a probability of 100%. An increase in the length of CNTs from 2 to 5 leads to an increase in the power of the percolation cluster at the same concentration of CNTs, for example, for a concentration of 0.35%, the power of a percolation cluster increases from 10% to 87%, and at a concentration of 0.3% for CNTs with a length of 2 the percolation cluster will not be formed, while for length 2.5 it will be formed, and increasing the length from 2.5 to 5 will increase the power from 10% to 80%.

To study the influence of the CNT diameter on the parameters of the percolation cluster, calculations were carried out for spherocylinders with length *l* = 2 and outer radius *R* = 0.125 in a cubic system of dimensions 25 × 25 × 25, their volume concentration varied from 0.3 to 1.0% (the number of spherocylinders was from 11,000 to 47,000), the inner radius varied from 0.025 to 0.004. An increase in the CNT radius by 60% (from 0.05 to 0.08 a.u.) leads to a 2.5-fold increase in the CNTs concentration (from 0.4 to 1.0%), at which a percolation cluster is formed with a probability of 100%. An increase in the CNTs diameter from 0.05 to 0.08 leads to a decrease in the probability of formation and power of a percolation cluster from 100 to 0% at a volume concentration of spherocylinders of 0.4%. The power of the percolation cluster also decreases; for example, at a concentration of 0.9%, an increase in diameter from 0.05 to 0.08 leads to a decrease in the power of the percolation cluster from 90% to 10%. An increase in the CNTs diameter can occur due to agglomeration and leads to an increase in the critical value of the CNTs concentration in the polymer at which a percolation cluster is formed.

To study the influence of the thickness of the CNTs interfacial layer on the parameters of the percolation cluster, calculations were carried out for spherocylinders with a length *l* = 4 and an inner radius *R*_0_ = 0.025 in a cubic system of dimensions 25 × 25 × 25, their volume concentration varied from 0.1 to 1.5% (the number of spherocylinders from 3000 to 30,000), the outer radius changed from 0.050 to 0.125. A 4-fold decrease in the thickness of the interfacial layer (from 0.100 to 0.025 a.u.) leads to a 3.7-fold decrease in the CNTs concentration (from 0.92 to 0.25%), at which a percolation cluster is formed with a probability of 100%. This leads to a decrease in the power of the percolation cluster; for example, for a volume concentration of 0.8%, the power of the percolation cluster decreases from 90% to 10%.

To study the influence of the geometric dimensions of the matrix on the parameters of the percolation cluster of CNTs, calculations were carried out for spherocylinders with a length *l* = 4 and an inner radius *R*_0_ = 0.025, an outer radius *R* = 0.125 in systems of the same thickness and different widths and lengths 25–250 × 25 × 25–250, the volume concentration of spherocylinders varied from 0.1 to 0.5% (the number of spherocylinders varied from 3000 to 350,000). The transition from an isotropic to an anisotropic system by a factor of 4 increase in the length and width of the system leads to a decrease in the concentration of CNTs, at which a percolation cluster is formed with a probability of 100%, from 0.25 to 0.22 and to a decrease in the power of the percolation cluster by 9.6 times (from 48 to 5%). A change in the ratio of linear dimensions to the thickness of the matrix also leads to a decrease in the concentration of formation and the power of the percolation cluster. As can be seen from Figure 2, an increase in the ratio of the length and width of the film to the thickness from 1 to 10 leads to a decrease in the power of the percolation cluster almost to zero.

These results show that particle agglomeration (an increase in particle diameter) leads to a decrease in the size of the percolation cluster. On the other hand, as the ratio of matrix thickness to particle size decreases, the size (strength) of the percolation cluster decreases. Agglomeration, as a result of which the particle size in the percolation cluster increases, leads to a decrease in the thickness and volume fraction of the percolation cluster. The permeability of polymer membranes with embedded carbon nanotubes will depend not only on the geometric dimensions of the CNTs and the thickness of the interfacial layer that forms between the CNTs and the polymer but also on the ratio of the geometric dimensions of the polymer matrix. The ratio of the sizes of the matrix and particles significantly affects the fraction of particles belonging to the percolation cluster, which is determined by the cluster power.

The experimental results of gas permeability [24] were calculated for the MMM model with the following parameters: matrix size 25 × 25 × 25 (film thickness 25 µm), spherocylinders length 2, and diameter *R*_0_ = 0.05, with shell thickness—0.100, the volume concentration of CNTs varied from 0 to 1.4% (number of spherocylinders from 0 to 50,000).

**Figure 3:**
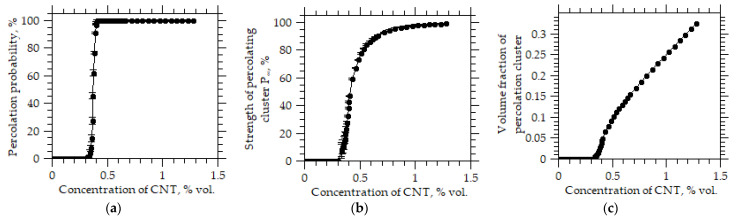
(**a**) Probability of formation of a percolation cluster (**b**) Strength of a percolation cluster (**c**) volume fraction of a percolation cluster from the volume concentration of CNTs.

Figure 3 shows the parameters of the percolation cluster. With the chosen parameters, the probability of the formation of a percolation cluster is equal to zero when the volume fraction of CNTs is less than *x* < 0.3, and when *x* > 0.4 it reaches 100%. In this case, the power of the percolation cluster and the fraction of the membrane occupied by the percolation cluster also change abruptly. Since the permeability of such membranes in (4) is determined by the volume fraction of $C_{x}$, a percolation cluster in the polymer matrix, the model allows one to choose the values of the permeability of gases for percolation regions (Table 2) and describe the observed experimental results.

**Figure 4:**
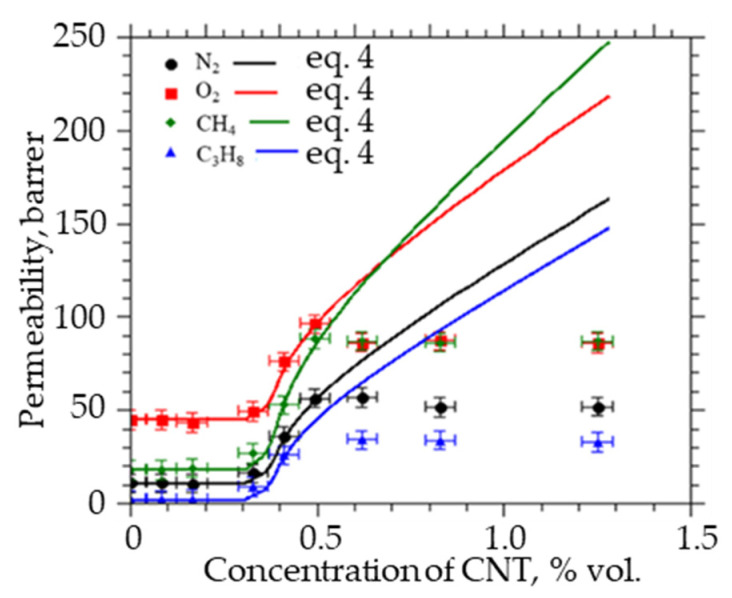
Permeability coefficients of nitrogen, oxygen, methane, and propane through PVTMS/CNTs membranes.

**Figure 5:**
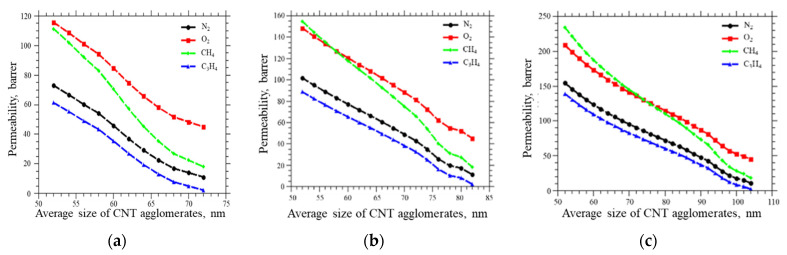
Gas permeability coefficients versus average diameter of CNT agglomerates at a CNTs volume concentration of (**a**) 0.62%, (**b**) 0.82%, and (**c**) 1.24%.

However, as can be seen from Figure 4, at fixed values of the permeability, with an increase in the concentration of particles (CNTs), there is a significant discrepancy between the model and experimental values of the permeability (Figure 5).

We assume that this is due to the agglomeration of CNTs in the polymer, which occurs due to the interaction between the side walls of CNTs with an increase in the concentration of particles, which leads to a decrease in the power of the percolation cluster at a given concentration of particles. Therefore, with increasing concentration, it is necessary to take into account the change in particle diameter and the corresponding decrease in the volume fraction of the percolation cluster and the permeability of the system.

For the previously obtained values of the permeability coefficients of PVTMS/CNT membranes, estimates were made of the change in the volume fraction of the percolation cluster with an increase in the average particle size (for single wall CNTs = 0.62, 0.82, and 1.24%) (Figure 5).

**Figure 6:**
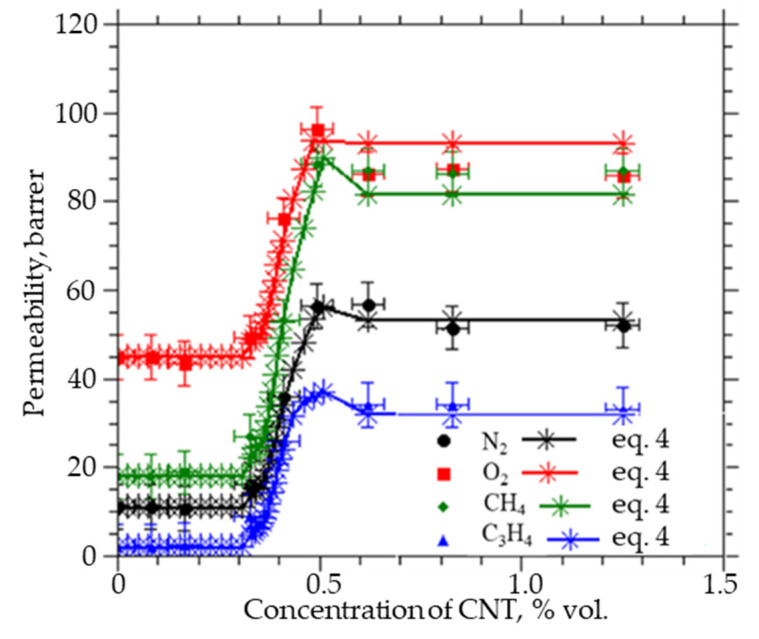
Permeability coefficients of nitrogen, oxygen, methane, and propane through PVTMS/CNTs membranes, taking into account agglomeration.

Figure 6 shows that an increase in the average diameter by 1.5–2 times (equivalent to an increase in the CNTs diameter from 52 to 100 nm) leads to a decrease in the permeability of all gases due to a decrease in the volume fraction of the percolation cluster. At the same time, the permeability coefficient of nitrogen, oxygen, methane, and propane through such membranes decreases by more than two times for all the studied gases. Taking into account the obtained modeling results, according to expression (4), the permeability of membranes at high concentrations was calculated (Figure 6). Within the error, the simulation results coincide with the experiment.

The calculated values of the gas permeability coefficients in such percolation systems correspond to the values of the diffusion coefficients of the studied gases over the CNTs surface calculated by the molecular dynamics method [37,38]. This result confirms our assumption about the mechanism of gas transport in such MMMs (Table 2).

## 4. Conclusions

In this work, we have shown that conventional models cannot describe the set of experimental data on the nonlinear change in the transport properties of MMMs with a change in the concentration of particles. These effects are sensitive both to the size of the matrix and to the characteristics of the introduced particles. In the model presented in this paper, the mass transfer of gases and liquids through polymeric membranes with embedded CNTs is considered as a parallel transport of gases through the polymer matrix and a percolation cluster—bound regions around the embedded CNTs. For the first time, a method for describing the transport characteristics of MMMs is proposed, taking into account the geometric characteristics of embedded particles and the finite dimensions of the matrix. The numerical simulation of such structures showed a significant change in the parameters of the percolation cluster with a change in the characteristic dimensions of the embedded particles and the linear dimensions of the matrix:

- An increase in the particle length leads to a decrease in the percolation concentration in a matrix of finite size. However, in this case, the power of the percolation cluster decreases significantly as does the fraction of the matrix in which the transport properties change. The combination of these effects leads to a decrease in the influence of the introduced particles on the properties of the matrix in the vicinity of the percolation threshold.
- An increase in the concentration of embedded particles leads to an increase in the probability of the formation of agglomerates and the characteristic size of the elements that make up the percolation cluster. In this case, the influence of individual particles decreases, and the characteristics of the percolation transition determine the ratio of the sizes of agglomerates and matrix. As the simulation showed, such an allowance for the increase in size makes it possible to describe the observed nonlinear changes in the permeability of the MMM, with the rest of the model parameters fixed. In addition to explaining the experimentally observed effects, this simulation makes it possible to describe the structure of the percolation cluster and MMM.
- An increase in the lateral linear dimensions of the matrix leads to a nonlinear decrease in the proportion of the matrix, which is affected by the introduced particles, and the transport characteristics of such MMMs deteriorate. Therefore, when scaling such systems, the conditions of the percolation transition and the structure of the percolation cluster will change. This effect must be taken into account when choosing embedded particles.

The obtained results allow us to take a different look at the effect of a nonlinear change in the properties of MMMs with an increase in the concentration of nanoparticles. In contrast to most models of transport in MMM, in which the main parameter is the concentration of embedded nanoparticles, our model has shown that the macroscopic changes are provided by the particles included in the percolation cluster. Therefore, when modeling the properties of such systems, it is necessary to focus on the parameters of the percolation cluster, taking into account the actual dimensions of the membrane and the introduced nanoparticles.

It should be noted that in this work we did not take into account the influence of the matrix on the percolation cluster of individual particles and considered only experimental data for MMMs with CNTs (both open and closed). Undoubtedly, the characteristics of the polymer and the interaction of nanotubes with the polymer are also essential for the properties of the MMM. The polymer can affect both the structure of the percolation cluster and the transport characteristics of the MMM. However, in this work, we studied the influence of only the geometric characteristics of the system in the region of the percolation transition. In this case, due to nonlinear changes in the region of the percolation transition, fluctuations in the macroscopic properties of such systems should be observed in such systems.

## Figures and Tables

**Table 1:** Percolation threshold for various geometric objects.

Source	Circle	Square	Segment	Sphere
[35,36]	1.122 [35]	0.982 [35]	5.637 [35]	0.032 [36]
Our software	1.127 ± 0.001	0.981 ± 0.001	5.636 ± 0.001	0.031 ± 0.01

**Table 2:** Experimental and calculated gas permeability coefficients and Knudsen diffusion coefficient for the studied gases.

Gase	*K_p_*, Barrer	*K_x_*, Barrer	*D_p_*, m^2^/s	*D_x_*, m^2^/s	*D_kn_*, m^2^/s
N_2_	11	429	3.8 × 10^−11^	1.52 × 10^−9^	~10^−6^
O_2_	44	507	7.6 × 10^−11^	8.8 × 10^−10^	~10^−6^
CH_4_	18	702	1.1 × 10^−11^	3.9 × 10^−10^	~10^−6^
C_3_H_8_	2	335	7.8 × 10^−12^	1.3 × 10^−9^	~10^−6^

## Data Availability

The data presented in this study are available on request from the corresponding author.
